# What Works? Strategies to Increase Reproductive, Maternal and Child Health in Difficult to Access Mountainous Locations: A Systematic Literature Review

**DOI:** 10.1371/journal.pone.0087683

**Published:** 2014-02-03

**Authors:** Abbey Byrne, Andrew Hodge, Eliana Jimenez-Soto, Alison Morgan

**Affiliations:** 1 Nossal Institute for Global Health, The University of Melbourne, Melbourne, Victoria, Australia; 2 School of Population Health, University of Queensland, Brisbane, Queensland, Australia; Aga Khan University, Pakistan

## Abstract

**Background:**

Geography poses serious challenges to delivery of health services and is a well documented marker of inequity. Maternal, newborn and child health (MNCH) outcomes are poorer in mountainous regions of low and lower-middle income countries due to geographical inaccessibility combined with other barriers: poorer quality services, persistent cultural and traditional practices and lower socioeconomic and educational status. Reaching universal coverage goals will require attention for remote mountain settings. This study aims to identify strategies to address barriers to reproductive MNCH (RMNCH) service utilisation in difficult-to-reach mountainous regions in low and lower-middle income settings worldwide.

**Methods:**

A systematic literature review drawing from MEDLINE, Web of Science, Scopus, Google Scholar, and Eldis. Inclusion was based on; testing an intervention for utilisation of RMNCH services; remote mountain settings of low- and lower-middle income countries; selected study designs. Studies were assessed for quality and analysed to present a narrative review of the key themes.

**Findings:**

From 4,130 articles 34 studies were included, from Afghanistan, Bolivia, Ethiopia, Guatemala, Indonesia, Kenya, Kyrgyzstan, Nepal, Pakistan, Papua New Guinea and Tajikistan. Strategies fall into four broad categories: improving service delivery through selected, trained and supported community health workers (CHWs) to act alongside formal health workers and the distribution of critical medicines to the home; improving the desirability of existing services by addressing the quality of care, innovative training and supervision of health workers; generating demand by engaging communities; and improving health knowledge for timely care-seeking. Task shifting, strengthened roles of CHWs and volunteers, mobile teams, and inclusive structured planning forums have proved effective.

**Conclusions:**

The review highlights where known evidence-based strategies have increased the utilisation of RMNCH services in low income mountainous areas. While these are known strategies in public health, in such disadvantaged settings additional supports are required to address both supply and demand barriers.

## Introduction

Geography can pose serious challenges to the delivery of health services and is a well documented marker of inequity in health outcomes [1]–[3]. Examples of hard-to-reach populations in low- and lower-middle income countries include remote island communities, sparely populated desert settlements and mountainous landscapes. The geographical challenges frequently result in very limited access to reproductive, maternal, newborn and child health (RMNCH) services and a higher risk of mortality [4]. The options for service provision, transport systems and cultural contexts differ substantially between these settings and demand careful consideration for effective responses. With many countries around the world pursuing the ambitious goal of universal health coverage, the risk exists that ‘hard to reach’ populations are left behind, unless health systems address the challenges of delivering services in disadvantaged locations [5]. This review focuses on mountain ranges of low- and lower-middle income settings where provision and coverage of reproductive, maternal, newborn and child health (RMNCH) services is a major public health challenge.

Barriers to health service utilisation have been categorised into access, availability, acceptability and cultural and traditional preferences, confidence in care and quality of services, health awareness and knowledge, and affordability [6], [7]. A review in 2009 of 82 studies confirmed that distance to health facilities is important, particularly in settings of isolation with a lack of transport, but in addition, socio-cultural factors, knowledge, economic situation and quality of care significantly influence a woman's ability to use health services [8]. Gabrysch and Campbell noted that these barriers frequently interact and further identified that groups in remote areas often face worse health infrastructure and transport, limited access to information, poverty and strong traditional systems [8]. Likewise, these factors influence the utilisation of antenatal care, family planning, and care for common childhood illnesses with greater impact on remote families [9], [10].

Significant mountain areas in many low- and lower-middle income countries represent the most extreme experiences of barriers to service utilisation. Actions with intensity proportionate to disadvantage may be required for such settings [11] as we aim for universal health coverage.

The purpose of this systematic literature review is to identify strategies to address barriers to RMNCH service utilisation in low resource, difficult-to-reach mountainous regions.

## Method

This systematic review is registered with PROSPERO through which the protocol has been peer-reviewed during development, number CRD42012003282 [12].

An ethics statement was not required for this work.

### Search

Literature was drawn from academic databases; MEDLINE, Web of Science, Scopus, Google Scholar, and Eldis up to 26 October 2012.

The search terms, applied with various boolean operators and medical subject headings (MeSH), are summarised in Table 1 with a full search strategy shown in Appendix S1. An iterative approach was used to refine the search strategies, systematic inclusion and exclusion criteria were applied using Endnote X5 software (Thomas Reuters, New York, USA) and a data extraction template using Microsoft Excel.

**Table 1:** Summary of search terms.

Separated by ‘OR’	AND	Separated by ‘OR’	AND	Separated by ‘OR’
maternal health; child health; newborn health; neonatal health; reproductive health; family planning; maternal mortality; child mortality; newborn mortality.		Afghan*; Bhutan*; Bolivia*; Burundi; Ethiopia*; Guatemala*; Indonesia*; Kashmir*; Kenya*; Ladakh*; Sikkim; Mongolia*; Morocc*; Nepal*; Pakistan*; Papua New Guinea; PNG; Rwanda*; Tanzania*; Tajikistan; Tibet*; Uzbekistan.		health service access*; health service utilis*; health service utiliz*; health service accept*; patient satisfaction; health service coverage; health service delivery; health care delivery.

### Eligibility criteria

Articles were included only if they; a) described testing of an intervention to overcome barriers to care-seeking, acceptability, satisfaction and/or utilisation of formal RMNCH services; b) took place in remote, sparsely populated mountain range setting above 1000 metres of low- and lower-middle income countries (see Table 1 for countries that meet this criteria) [13]; with c) study designs of trials (randomised, cluster or community), cross sectional, case study or systematic review. Narratives, opinion pieces, commentaries were excluded. Detailed criteria may be viewed in Appendix S2.

### Study selection

From 4,130 articles, 34 studies were selected. To determine if studies were set in mountain ranges, rural or remote, and at altitude above 1000 m, National Geographic topographic global maps, satellite terrain maps, and country description documents were examined [14]–[16]. The final step in the refinement process involved determining whether or not the study's location was a mountainous/highlands region, or a lowland or plateau. For this purpose, a web search was undertaken using Google and the names of the districts/sites along with keywords such as ‘topography’, ‘terrain’, ‘mountainous’, ‘highlands’, ‘lowlands’ and ‘plateaus’. This search was independently undertaken by two of the authors and disagreements were resolved by consensus. The process of article selection is presented in Figure S1.

### Data extraction

Data extraction was undertaken independently, reviewing full text papers, by four researchers using a piloted and revised spreadsheet. Finally, one researcher reviewed all inclusions and inconsistencies, which were minimal, were resolved in discussions between the four researchers.

Data was extracted for: a) setting and population; b) relevant RMNCH service; c) intervention description; d) strengths/successes; e) weaknesses/challenges to the intervention identified by the study ; and f) quality (study design and limitations in method and analysis).

### Quality of evidence in individual studies

The quality of individual studies was assessed using the Effective Public Health Practice Project (EPHPP) Quantitative Assessment Tool [17] and the Critical Appraisal Skills Programme (CASP) qualitative appraisal tool [18] for quantitative and qualitative studies respectively into the data extraction spreadsheet. For quantitative studies consideration was given to selection, study design, confounders, blinding, data collection, withdrawals and drop-outs, intervention integrity and analysis [17]. Studies were ranked as weak (further research is very likely to have an important impact on our confidence in the estimate), moderate, or strong (further research is unlikely to change our confidence in the estimated effect). Qualitative studies were appraised on ten elements considering aims, design, method, data collections, roles and biases, ethics, analysis, validation and value [18].

### Summary measures

The predominant summary measures of results are the proportion of change comparisons of before-after or intervention-control, odds ratio (OR), or relative risk (RR).

### Synthesis of results

The diversity of experimental designs used and strategies examined by these studies precludes a full meta-analysis and quantitative evaluation. Instead, we opted for a qualitative analysis identifying broad conclusions and present a narrative review of the key themes arising from the literature. Extracted data regarding the strategy used, quantitative results and author conclusions were used to group studies according to similarities in structure and outcomes.

## Results

### Study characteristics and quality

The search strategy yielded evidence for inclusion from 11 countries; regions of Afghanistan, Bolivia, Ethiopia, Guatemala, Indonesia, Kenya, Kyrgyzstan, Nepal, Pakistan, Papua New Guinea and Tajikistan. The majority of studies were cross-sectional (19) followed by experimental trials (12), case studies (2), and a case-control study (1). The quality ranks of quantitative studies (n = 29) was evenly spread across strong (8), moderate (13) and weak (8) although the evidence is heterogeneous and best judged for individual studies rather than pooled.

The results, characteristics and quality features of individual included studies are shown in Appendix S3.

Strategies identified in the 34 studies can be divided into four broad categories: delivery of services to remote communities; improvements to the desirability of existing services through improved facility and provider performance; improving affordability of services; generation of demand for services and improving acceptability; and improving health knowledge for timely and appropriate care-seeking. Examples are summarised in Table 2.

**Table 2:** Summary of strategies identified to improve utilisation of RMNCH services in low and lower-middle income mountainous settings.

**Taking services to the community for accessibility and acceptability**	Community health workers have been most effective through rountine monthly home visits to all households for preventive and certain curative services, particularly for family [19]–[24]. Success is linked with regular supplies, on-site training and supervision. The influence of small renumeration or recruitment criteria is unclear.
	Task-shifting has increased availability and acceptability of family planning services and emergency obstetric care. Services provided by lay health workers have been preferred over formal health workers by communities. Engagement with community representatives, leaders and village committees is an important accompaniment [25]–[29].
	Home-based administration of critical drugs such as misoprostol by CHWs has reduced incidence and severity of complications with high safety [30].
	Mobile camps have provided female sterilisation of equivalent quality to hospitals although their delivery has been infrequent in mountain areas [31].
	Maternity birthing homes linked by radio to clinics increased skilled delivery care however have been difficult to sustain [32].
	Telemedicine has connected health workers and patients in mountain villages with doctors in central hospitals to increase utilisation and satisfaction [33].
**Increasing desirability by improving facility and provider performance**	Upgrading facilities with equipment, guidelines, training and supervision has reduced mortality with follow-on increased utilisation [34]. Integrating services into one session is more efficient given the difficulty for users to repeatedly access facilities [35].
	Coaching providers for effective communication and sensitive care through training and orientation from community leaders and lay providers increases staff motivation, quality of care, referral rates and consumer satisfaction [32], [36].
	Training and supervision through distance learning programs, job aids, on-site and off-site supervisor contact, and supervision aids improves staff motivation and performance and in turn utilisation [38]–[40].
**Increasing affordability of using existing services**	Free maternity care increases care-seeking and reduces delays by overcoming concerns of inability to pay or loan acquisition [41].
	Financial stimulants for demand, provider performance and facility compensation have increased facility-based births although affordability and other barriers persist; travel costs, reach of information, funding flows [42], [44].
**Improving demand through participation and engagement**	Community planning through facilitated, structured, action-oriented women's groups has increased health knowledge, care-seeking and practices and service utilisation [32], [45]–[48].
	Engaging traditional healers and clearly defining roles within health services has increased referrals to facilities [32], [49], [50].
**Increasing health knowledge to motivate care-seeking**	Awareness-raising through intergenerational community discussions, mentor groups for youth, and community facilitators has improved gender equity, health knowledge and service use. Impact is linked with skills of community agents and male involvement [51]–[53].
	Information tools, such as cards, posters, and charts have improved health knowledge and service utilisation in some settings although in other instances knowledge has not translated to action, particularly owing to cultural barriers [32], [39]. Social marketing has been an effective tool to raise awareness and service use [54].

### Taking services to the community

Providing **community health workers** (CHW) with small salaries, on-site monthly training and supplies package, and fortnightly supportive supervision has led to coverage of at least one monthly health visit for every household in Pakistan [19]. This evidence refers to CHWs who are a lay cadre, not formal health professional with academic education, but given varying degrees of training and typically recruited from within the community which they serve. In mountainous Mastang of Baluchistan province the Lady Health Worker (LHW/CHW) program was able to increase the proportion of pregnant women delivered with a skilled birth attendant (SBA) to 51%, compared to the national average of 39% [20] and there was a 50% greater likelihood of women using contraceptives (OR 1.5, 95% CI 1.0–2.1, p = 0.031). In the mountainous North West Frontier and FATA areas coverage of family planning was high regardless of the LHWs although in AJK and Northern Areas, also in mountain ranges, contraceptive use was directly linked to the LHW program [21]. The approach coincided with a halving of both the infant mortality rate (from 130 deaths per 1,000 live births to 64), and the maternal mortality ratio (from 596 deaths per 100,000 live births down to 246) [19], although the before and after cross-sectional study design means these results could be coincidental. CHWs do not necessarily require cash remuneration for success as demonstrated in Nepal with a 41% increase in care-seeking for child illness through the actions of their Female Community Health Volunteers and a 28% reduction in under-5 deaths (RR 0.72, 95%CI 0.63–0.82) from a high quality trial [22]. However, recruiting LHWs (or CHWs) to serve in disadvantaged areas has been challenging with 35% vacant posts persisting in Pakistan [19]–[21] and in Guatemala this type of model has been more expensive than standard rural primary healthcare services [23]. In their large controlled trial in Tigray, Ethiopia, Ali *et al* reported that CHWs have only been able to motivate those living near to facilities to use services highlighting the overlap of knowledge and access barriers, although they did demonstrate a 34% reduction in under-5 deaths through combined improved care-seeking and preventative child care practices (RR 0.66, 95%CI 0.46–0.95) [24].

**Task-shifting** has increased availability and acceptability of services. Auxiliary nurse-midwives trained to provide intrauterine contraceptive device (IUDs) services increased IUD users from 18 to 71 per month with only 0.6% complications in rural Guatemala [25]. In Ethiopia Community-Based Reproductive Health Workers (CBRHWs) needed only 10 days of theory plus clinic-based training to safely provide injectable contraceptives. This served a new population; 41% of users had never used modern contraceptives and more women who were older, unmarried, greater parity, of low education used contraceptives from CBRHWs (p<0.05) [26] in preference to more senior clinic-based Health Extension Workers (p<0.05) [26], [27]. Similarly this model in Tormay and Farza of Afghanistan, combined with education for community and religious leaders, increased the contraceptive prevalence rate (CPR) in only 8 months from 24 to 51% and 9 to 34%, respectively, in contrast to the control where CPR rose only moderately from 16 to 26% [28].

Task-shifting of comprehensive emergency obstetric care (CEOC) to non-physician clinicians (nurse-equivalents) through 9 months of training covered 63% of CEOC cases in Ethiopia where CEOC facilities were present (1: 350,000 population) yet staffed with only 4 surgical physicians [29]. Non-physician clinicians provided more emergency caesareans while physicians were more inclined to perform elective caesareans (p<0.001) with equivalent quality [29]. Most effective task-shifting approaches involve engagement with community representatives, leaders and local or village health committees [26], [27].

**Home-based administration** of misoprostol by CHWs attending home deliveries in a large community-based controlled trial of Ethiopia reduced the incidence of post-partum haemorrhage by 58% (OR 0.42, 95%CI 0.25–0.61) and lowered severity with high safety [30].

**Mobile camps** providing permanent female contraception (sterilisation) have high satisfaction reported by clients with equivalent quality to hospitals although are rarely offered in the Mountains ecoregion of Nepal compared to the plains (Terai) (p<0.05) [31].

**Maternity birthing homes** in West Java, Indonesia, staffed with SBAs linked by radio to clinics increased skilled delivery care from 17 to 36% (p<0.05) however the study offers only weak evidence and sustainability was an issue with two of ten homes closed within two years [32].

**Telemedicine** with computers and wireless internet has connected health workers and patients in the mountain villages of Myagdi district, Nepal, with central hospitals to increase access to and utilisation of specialist doctors. Patients reported increased satisfaction and confidence in care [33].

### Improving facility and provider performance for quality and satisfaction

**Upgrading facilities** with functional neonatal care equipment accompanied by training, guidelines and supervision for nursing staff has been linked with 44% lower neonatal mortality (RR 0.56, 95%CI 0.45–0.69), largely averted pneumonia deaths, and a minor increase in service utilisation in only two years in the highlands of Papua New Guinea [34].

**Integrating services** by adding to or merging existing services has increased coverage where travel and access is challenging and points of contact are few. Moderate to strong evidence from various mountain range settings of Kenya shows that integrating HIV care with family planning, or with antenatal care services, increases uptake of HIV testing and treatment among women by up to 25%. Integrated care consistently is more time demanding however satisfaction among HIV-positive women was very high regardless of waiting times [35].

**Coaching health workers for effective communication, responsive, and sensitive care** through ‘Professional Attitude and Effective Communication’ skills training with orientation from community leaders and lay providers increased provider motivation and consumer satisfaction. Partner attendance for facility-based deliveries (FBDs) rose from 10 to 70%, and harmful practices reduced by 90%, with 30% fewer delivery and caesarean complications in Tajikistan and Kyrgyzstan [36], [37]. In Guatemala a similar approach improved collegiality between health workers and traditional birth attendants (TBAs) and was associated with fewer perinatal deaths among TBA referrals, from 22 to 12% (p<0.05) [32]. However, each of these models requires higher quality evidence.

**Training and supervision** through distance learning programs and job aids improves staff motivation and performance in remote areas. In Guatemala this improved the quality of family planning counselling and in turn utilisation by 12% (p<0.004) [38], [39]. Vernon *et al* reported that the trial of supervision through a combination of supervisor visits and full-day district-level meeting was more preferred by remote health workers, less expensive, and increased CPR more effectively (9.7% compared to a 22% decrease and 6.5% increase, respectively) compared to only supervisor visits or self-assessment workshops [40].

### Financial initiatives to ease the burden of service fees and costs of care-seeking

**Free maternity care** has reduced care-seeking delays in Nepal with fewer families hesitating due to inabilities to pay or acquiring loans. This has aligned with increased FBD for normal births by 19%, complications by 15% and caesarean by 18% although the cross-sectional study precludes assertion of causation [41].

**Financial stimulants** for demand, provider motivation and facility compensation, applied in Nepal's Safe Delivery Incentive Programme coincide with increased FBDs with those exposed being 24% more likely to have FBD. However, affordability and other barriers persist: in 2008 the FBD incentive covered only 30–50% of the average travel costs in mountain districts and promotion was challenging in the sparsely populated mountains where women with no education or of *Dalit* caste were least likely to be informed [42], [43]. With revisions the program has become more effective with less complexity, full orientation of program managers, and effective fund disbursement procedures [42]; and in 2010 awareness was high nationally, with significant distribution to the lowest wealth quintile [44].

### Community input for demand generation and culturally-appropriate health services

**Community-led planning** activities have improved health knowledge, care-seeking, service utilisation and perinatal mortality in multiple sites however models are varied and overall the evidence from mountain range settings is not drawn from high quality controlled trials. Facilitated structured planning and action oriented women's group sessions moving from problem analysis, strategies, implementation and review in Bolivia, coincided with a decrease in perinatal mortality from 117 to 43 deaths per 1000 live births (p<0.001) and increased participation in women's organisations from 7 to 54% (p<0.001) [45]. In other sites of Bolivia this model led to 73% of participants expressing willingness to use reproductive health services [46], CPR increased from an estimated zero to 27%, clean delivery kits were newly introduced and used in 27% of homebirths, and early breastfeeding increased from 25 to 57% within 3 years [32]. In two projects, inclusion of health, education and agricultural representatives and motivation of community leaders in planning activities led to perceived improvements in community-wide improved health behaviours and increases in health service utilisation in Papua New Guinea [47], [48] although this was not quantified or validated.

**Engaging traditional healers** and clearly defining roles with them, has reduced delays in Guatemala with early referrals by TBAs for complicated births up from 26 to 36%, and non-complicated births from 5 to 21% [32]; while in Taplejung, a mountain district of Nepal, this strategy was perceived to have doubled the attendance at some health facilities [49], [50].

### Knowledge and awareness-raising to motivate care-seeking and utilisation

**Awareness-raising** through intergenerational community discussions, mentor groups for youth, and literacy and livelihood training for women in Ethiopia diminished harmful traditional practices, increased school enrolment for girls aged 10–14 from 71 to 96% (p<0.05), improved reproductive health and family planning knowledge (p>0.001), and rose CPR among sexually active girls from 43 to 74% (p>0.001) [51]. Male involvement in reproductive and women's health messages has been a defining feature for change;

*“they (men) even encourage us to use contraceptives and remind us if we have taken the pill”* (woman in focus group discussion, Ethiopia) [52].

Shanker and colleagues (2009) present high quality evidence of community facilitators, recruited from local communities in Indonesia, displaying high levels of knowledge, care and thoroughness in their work, were able to motivate pregnant women for 85% compliance with micronutrient supplementation and increase in SBA utilisation from 35 to 53% with a resultant 33% reduction in early infant mortality (RR 0.67, 95%CI 0.49–0.92, p = 0.0117) [53].

**Information tools**, such as cards, posters and charts, improved health knowledge and increased use of contraception services from 31 to 34 clients per day in Guatemala [39]. However, in Indonesia, knowledge did not always translate to action: only 10% of high risk and 27% of very high risk women had FBDs as advised based on their home visit with CHWs using antenatal risk assessment cards [32]. Social marketing was a powerful tool for increased awareness as well as actual utilisation in rural Meru and Kirinyaga of Kenya. Based on formative investigation, a new condom brand was released with appearance, price and messages designed to suits the setting. With radio announcements, cinema shows, and a mobile van with education and free samples, awareness of family planning for all methods rose from 64 to 81% and utilisation increased from 21 to 35%, predominantly of condoms, in only one year [54]. Engagement with local retailers was considered critical to success as they are key information sources for consumers.

## Discussion

Delivering quality health care services to populations in hard-to-reach environments is challenging. There are some promising strategies that can address the burden of geographical inaccessibility. Task shifting, strengthened roles for CHWs and volunteers, mobile teams, incentives and removal of user fees, local awareness initiatives, and highly inclusive structured health promotion and planning forums have proved effective in a variety of difficult to reach mountainous settings in broadening both the scope and reach of RMNCH services. These are not novel strategies, and each has a proven efficacy in more accessible settings [6], [55]–[59]. Our review highlights which strategies also work in more difficult contexts.

Geographical barriers limit the scope of health research that is conducted in these settings, creating further inequity in our understanding of the true barriers, or the potential strategies that may work. A possible limitation of this review is the shortage of evidence; studies concerning health services are not available for all relevant low resource mountain range settings of the world however this review does include 11 countries and should be considered representative of the context. In many cases the evidence for a particular strategy is drawn from one setting only. It is also likely that approaches that are successful in more accessible areas may well work in mountainous areas however setting-specific evidence is lacking. Of particular note is that while there exists many examples of strategies to take health services to the community, our search did not yield any such studies. Improved transport is a recognised means for increasing timely access to care, however examples of successful programs are largely based in non-mountainous settings. Air retrievals are notoriously difficult in mountainous terrain and initiatives that are promising in other settings, such as stretcher transport teams or other local initiatives, are not reported from the - very settings where they may be of most value. Additionally, there is evidence of the benefits of comprehensive, regular outreach teams for increased service coverage and facility-based care uptake however the evidence is not specifically available from remote high altitude sites [60]–[62]. Maternity waiting homes (MWH) have been introduced in many settings to overcome the geographical access barriers to women having skilled birth attendance, but with very mixed results [56] and there was only one weak MWH study that met the inclusion criteria in our review. Further research on strategies to increase access to existing distant services is required.

From the available reviews however it appears that the most successful programs look beyond the tyranny of distance to also address the greater contribution of other utilisation barriers; that is strongly held cultural beliefs, greater socioeconomic disadvantage and lower levels of health knowledge. The reach of services must be extended through selected, trained and supported CHWs who team with formal health workers, task-shifting to peripheral cadres, home-based administration of medications to buy time for care-seeking. Facilities must be equipped with supplies and staff to deliver quality, reliable and culturally sensitive care and integrated services may be more efficient when users must traverse the mountains to reach providers. Health workers may be better able to provide this through innovative training and supervision solutions and support from peers and community leaders. With structured, facilitated input and health promotion that reaches youth, women, men and elders, communities may be able to self-mobilise for greater care-seeking. We need to recognise that ‘more of the same’ will not bring the health improvements that we seek for women and children in these difficult settings. We must concentrate efforts to increase the evidence of what works, and to deliver what we know can be effective.

## Supporting Information

Figure S1 **Flow diagram of article selection process.**
(PDF)

Appendix S1 **Full search strategy of one database.**
(DOC)

Appendix S2 **Inclusion and exclusion criteria.**
(DOC)

Appendix S3 **Summary of the characteristics and quality of included studies.**
(DOC)

Checklist S1 **PRISMA Checklist.**
(PDF)
