# Supplementary figures and images for: What Works? Strategies to Increase Reproductive, Maternal and Child Health in Difficult to Access Mountainous Locations: A Systematic Literature Review
Source: PLoS One. 2014 Feb 3;9(2):e87683. doi: 10.1371/journal.pone.0087683 (PMC3912062; doi:10.1371/journal.pone.0087683)

Figure S1. Process of article selection

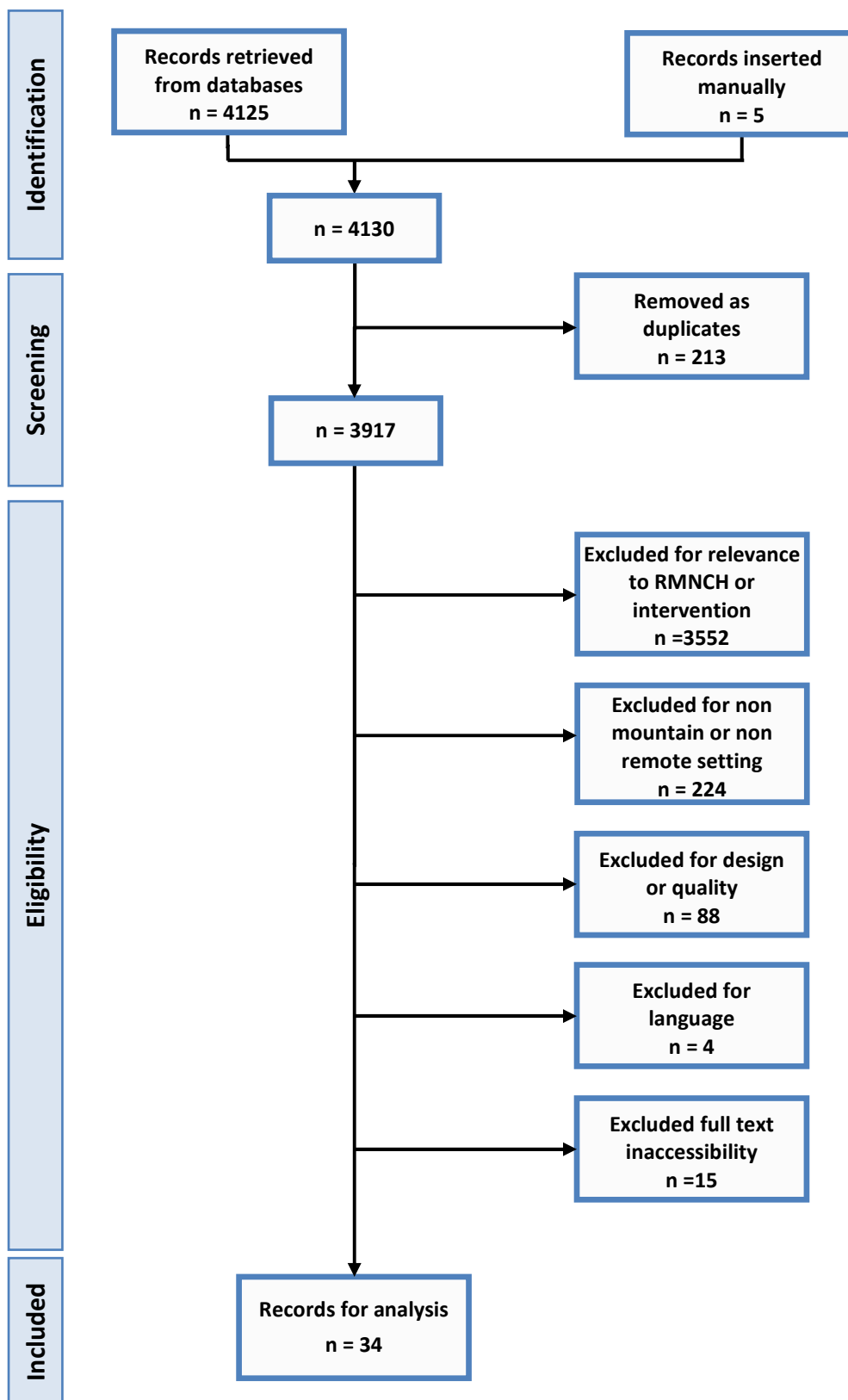

Supplement: Figure S1 — Flow diagram of article selection process. (PDF) [file pone.0087683.s001.pdf]
