# Supplementary material for: What Works? Strategies to Increase Reproductive, Maternal and Child Health in Difficult to Access Mountainous Locations: A Systematic Literature Review
Source: PLoS One. 2014 Feb 3;9(2):e87683. doi: 10.1371/journal.pone.0087683 (PMC3912062; doi:10.1371/journal.pone.0087683)
Supplement: Appendix S1 — Full search strategy of one database. (DOC) [file pone.0087683.s002.doc]

## Web Appendix S1. Full search strategy of one database

| **Medline** | Topic=(((((((((((((((Afghan*) OR Bhutan*) OR Bolivia*) OR Burundi*) OR Ethiopia*) OR Guatemala) OR Indonesia*) OR Kashmir*) OR Kenya*) OR Ladakh) OR Sikkim) OR Mongolia*) OR Morocc*) OR Nepal*) OR Pakistan*) OR "Papua New Guinea") OR "PNG") OR Rwanda*) OR Tajikistan*) OR Tanzania*) OR Tibet*) OR Uzbekistan*) |
| --- | --- |
|  | AND [[Topic=((((((((("Maternal Health") OR "Child Health") OR "Newborn Health") OR "Neonatal health") OR "Reproductive Health") OR "Family Planning") OR "Maternal Mortality") OR "Child Mortality") OR "Newborn Mortality") OR "Neonatal Mortality")] OR [[MeSH Heading:exp=(((((((Maternal Health Services) OR Child Health Services) OR Reproductive Health) OR Women s Health) OR Infant Newborn) OR Maternal Mortality) OR Child Mortality) OR Family Planning Services)]] |
|  | AND [[Topic=((health) service access* OR health service utilis*) OR health service utiliz*)] OR [MeSH Heading=((((((Patient Acceptance of Health Care) OR Attitude to Health) OR Patient Satisfaction) OR Health Services Accessibility) OR Quality of Health Care) OR Delivery of Health Care) OR Delivery of Health Care Integrated)]]. Refined by: [excluding] MeSH Qualifiers =(CEREBROSPINAL FLUID OR ISOLATION PURIFICATION OR ENZYMOLOGY OR VETERINARY OR POISONING OR ULTRASTRUCTURE OR CHEMICAL SYNTHESIS OR PHARMACOKINETICS OR ANALOGS DERIVATIVES OR RADIONUCLIDE IMAGING) AND [excluding] Research Areas=(METEOROLOGY ATMOSPHERIC SCIENCES OR ONCOLOGY OR COMPUTER SCIENCE OR CARDIOVASCULAR SYSTEM CARDIOLOGY OR ALLERGY OR NEUROSCIENCES NEUROLOGY OR BUSINESS ECONOMICS OR UROLOGY NEPHROLOGY OR OTORHINOLARYNGOLOGY OR PSYCHIATRY OR ENERGY FUELS OR GERIATRICS GERONTOLOGY OR ZOOLOGY OR AGRICULTURE OR AUDIOLOGY SPEECH LANGUAGE PATHOLOGY OR NUCLEAR SCIENCE TECHNOLOGY OR ORTHOPEDICS OR SUBSTANCE ABUSE OR BIOCHEMISTRY MOLECULAR BIOLOGY OR OPHTHALMOLOGY OR VETERINARY SCIENCES OR ARCHITECTURE OR ENVIRONMENTAL SCIENCES ECOLOGY OR ANATOMY MORPHOLOGY OR BIOPHYSICS OR PLANT SCIENCES OR METALLURGY METALLURGICAL ENGINEERING OR OPERATIONS RESEARCH MANAGEMENT SCIENCE OR DENTISTRY ORAL SURGERY MEDICINE OR PHYSICS OR RESEARCH EXPERIMENTAL MEDICINE OR RHEUMATOLOGY) AND [excluding] Publication Types=(NEWS OR EDITORIAL OR BIOGRAPHY OR LETTER OR GUIDELINE OR LEGISLATION OR COMMENT) AND [excluding] Research Areas=(GENETICS HEREDITY) AND [excluding] Research Areas=(TOXICOLOGY OR CRIMINOLOGY PENOLOGY OR GEOLOGY OR ENTOMOLOGY) |
