# Supplementary material for: What Works? Strategies to Increase Reproductive, Maternal and Child Health in Difficult to Access Mountainous Locations: A Systematic Literature Review
Source: PLoS One. 2014 Feb 3;9(2):e87683. doi: 10.1371/journal.pone.0087683 (PMC3912062; doi:10.1371/journal.pone.0087683)
Supplement: Appendix S2 — Inclusion and exclusion criteria. (DOC) [file pone.0087683.s003.doc]

## Web Appendix S2. Inclusion and exclusion criteria.

| **Inclusion** **criteria:**  Publications that meet all of the following criteria: | |
| --- | --- |
| **1.** | Describe the testing of an intervention to address barriers to care-seeking, acceptability, satisfaction and/or utilisation of formal reproductive maternal, newborn and child health care services |
| **2.** | Set in remote or rural, sparsely populated mountain ranges at altitude above 1000m |
| **3.** | Low- and lower middle-income countries only [13] |
| **4.** | Quantitative or qualitative studies |
| **5.** | Are of the following study designs: trials (randomised, cluster or community), systematic review, cross sectional, case study, literature review |
| **Exclusion** **criteria:** | |
| **1.** | Report on services other than those for reproductive, maternal, newborn or child health |
| **2.** | Do not demonstrate a clear research design or methodology, ie. commentary, opinion, editorial |
| **3.** | Published in a language other than English |
| **4.** | Full text documents are not accessible |
