# Supplementary material for: What Works? Strategies to Increase Reproductive, Maternal and Child Health in Difficult to Access Mountainous Locations: A Systematic Literature Review
Source: PLoS One. 2014 Feb 3;9(2):e87683. doi: 10.1371/journal.pone.0087683 (PMC3912062; doi:10.1371/journal.pone.0087683)
Supplement: Appendix S3 — Summary of the characteristics and quality of included studies. (DOC) [file pone.0087683.s004.doc]

## Web Appendix S3. Summary of the characteristics and quality of included studies.

| **Action** | **Reference** | **Setting** | **Intervention description** | **Outcomes**  **(Odds ratio (OR), Relative risk (RR) proportion change)** | **Quality** | | |
| --- | --- | --- | --- | --- | --- | --- | --- |
|  |  |  |  |  | **Study design, method, population** | **Limitations identified in appraisal** | **Quality rating** |
| Taking services to the community for accessibility and acceptability | | | | | | | |
| **Community health workers** | Ali, M. et al. (2005). [24] | Ethiopia. Tigray region | Community facilitators (local mothers) trained for promotion of child health and development, prevention, home care, care-seeking and compliance, to recognise key signs and symptoms of important childhood illnesses. | FBDs increased among those living near facilities but not the target groups; unmarried parents and older fathers. Risk of death in children under 5 years was 0.66 (95%CI 0.46–0.95) for the intervention compared to control. | Controlled trial. Intervention/ control. Before/ after. 8498 children under 5 years of age. |  | Strong |
|  | Barzgar, MA. et al. (1997). [19] | Pakistan. Mastang, Balochistan province. | Lady Health Worker (LHW) program; community women provide RMNCH services. Have grade 8 schooling, in-the-field training monthly with supply packages, supervision fort-nightly. One LHW per 150 households, 5 home visits per day, visit every house monthly. | Infant mortality rate down from 130 to 64 per 1000 live births. Maternal mortality ratio (MMR) from 596 to 246 per 100,000 live births. FBDs from 11.8 to 17.5%; 12-23 month olds full immunisations in Mastang up from 26 to 94%. | Cross-sectional. Before/after. Quantitative. Staff monthly records. 25365 records of child morbidity (under 5 years). | Strong effect. Indirectness; analysis details not presented.  Analysis not accounting for confounders. | Moderate |
|  | Douthwaite, M. et al. (2005). [21] / Hafeez, A. et al. (2011). [20] | Pakistan. National, area disaggregation for North-West Frontier (NWF), FATA and Northern. | As per Barzgar, MA et al. (1997). | 50% greater likelihood of contraceptive use by women served by LHWs; OR 1.5 (95% CI 1.04-2.16, p=0.031) Shortage of recruits. Education criteria exclude disadvantaged women. Mountainous NWF and FATA have high coverage regardless of LHWs. However in mountainous AJK and Northern Areas contraceptive use was linked to the LWHs. | Cross-sectional. Intervention/ control. PPS and random sampling 4277 women married, rural, 15-49 years. | Strong effect. Controlled for socio-economic confounders. | Moderate |
|  | Fort MP et al. (2011). [23] | Guatemala. Bocacosta and Ostuncalco | Community Auxiliary Nurses trained full-time for 6 months for preventive and curative care and health promotion. Work in teams of 2-4 at community health clinics serving 9,000 population plus routine home visits delivering RMNCH package. | Bolacosta and Ostuncalco respectively; full vaccinations at 23 months rose from 48 to 96% and 44 to 92% (p<0.001), ANC3+ from 27 to 72% and 16 to 62%, PNC within 24 hrs from 29 to 32% and 5 to 32%. More costly than standard; US$19-27/capita vs US$8. | Cross-sectional. Before/after.  Census database records, 3002 families | Large sample, analysis for confounders and significance with strong effect | Moderate |
|  | Pandey, MR. et al. (1991). [22] | Nepal. Jumla district | Female Community Health Volunteers (FCHVs) home visits fortnightly with anti-biotics for pneumonia in children <5 years. No referrals possible as facilities are largely inaccessible. 1 FCHV per 1000 population, average 30 minutes to each household. | FCHVs visit average of 15 households daily, detect 80% of estimated pneumonia cases. Mother’s seeking FCHVs’ care increased from 15 to 56%. Resultant 28% reduction in risk of all causes under-5 deaths (RR 0.72, 95%CI 0.63-0.82). | Controlled trial. Intervention/ control. Purposive selection. 6684 children under 5 years. | Matched. Controlled for confounders. Mortality tracked monthly. | Strong |
| **Home-based administration** | Prata, N. et al. (2009). [30] | Ethiopia. Tigray province | Home administration of prophylactic oral misoprostol 600mcg through community birth attendants (highly respected TBAs). | Fewer cases of excessive bleeding (>500mL); 8.9 vs 18.9% (OR 0.42 (95%CI 0.25-0.61). Of post-partum haemorrhage cases, blood transfusion needed by 1% compared to 6.2% of controls (OR 0.13, 95%CI 0.04-0.36). | Community controlled trial. Intervention/ control. 1000 home deliveries by TBAs. | Controlled for confounders. Sample selection not detailed. | Strong |
| **Task-shifting** | Argaw, DM. et al. (2007). [27] | Ethiopia. South Gondar in North West. | Community-Based Reproductive Health Workers (CBRHWs), who are unpaid lay health workers, tasked to distribute pills, condoms and reproductive health education to the community. | Higher CPR with higher awareness of (OR 2.32, 95%CI 1.75-3.08), acceptance of (OR 6.63, 3.59-12.43) and talking with CBRHAs (OR 5.05, 3.22-7.96). Low CPR areas had low participation in CBRHW selection, low motivation and weak supervision of CBRHWs. | Cross-sectional. Comparison of project areas.  792 women for quantitative. Interviews, FGDs. | Controlled for confounders. PPS and random sampling. Strong effect. | Moderate |
|  | Gessessew, A. et al. (2011). [29] | Ethiopia. Tigray province | Transferring comprehensive emergency obstetric care (CEOC) skills to 11 non-physician clinicians (NPC) through 6-9 months of training. Area had CEOC facilities (1: 350,000 population) but only 4 surgical physicians. | NPCs attended to 63% (p<0.001) of all obstetric procedures providing care of equivalent quality to the physicians. Caesareans by NPCs were more often for emergency while those by physicians were often elective (p<0.001). | Cross-sectional. Review of records. 25,629 deliveries, 11,059 obstetric procedures in 13 facilities. | Controlled for confounders. Analysis for statistical significance. | Moderate |
|  | Huber, D. et al. (2010). [28] | Afghanistan.  Tormay and Farza. | Information and education for women, men, CHWs, clinicians and religious leaders plus injectable contraceptives (Depo-Provera®) newly delivered by volunteer CHWs. | Increased CPR; from 24 to 51% in Tormay and 9 to 34% in Farza, over 8 months. In contrast, 13 provinces with standard services changed only slightly from 16 to 26%. | Cross-sectional. Before/after. Lot Quality Assurance Sampling (LQAS). | No analysis of statistical significance or confounders. | Weak |
|  | Prata, NA. et al. (2011). [26] | Ethiopia. Tigray province | Injectable contraceptives by newly trained Community-Based Reproductive Health Workers (CBRHWs) - unpaid CHWs given 10 days of theory plus 2-stage clinical training. In contrast to more senior clinic-based Health Extension Workers (HEWs).. | 41% were new users (p<0.05). Discontinua-tion after 3 injections 2% for CBRHAs and 11% for HEWs. Women preferred CBRHWs. CBRHWs generated uptake in more women who were older, unmarried, had greater parity, and were lesser educated (p<0.05). | Non-randomised community trial. Intervention/ control. 1,062 women | Controlled for confounders. Analysis for statistical significance. | Strong |
|  | Vernon, R. (2009). [25] | Guatemala. Chinquimula,  Escuintla  Suchitepequez,  Chimaltenango, Jalapa, Jutiapa, Progreso, Santa Rosa, Solola | Task-shifting of intrauterine device (IUD) services to auxiliary nurses at rural health posts. 2-days theory and practical on-site training. Shift in political support against family planning saw course completion rate fall; 85% in first phase to 36% in second. | Number of IUD users rose from 18 to 71 per health centre per month. Pills, condoms and injection users decreased by 10%. High quality; one laceration and one pregnancy out of 301 users in one month. After 2 years 80% of nurses continued to provide IUD services. | Cross-sectional. Before/after. Review of records. 103 health centres | Quality; adverse events, satisfaction, discontinuation not validated among users. | Weak |
| **Mobile camps** | Thapa S. et al. (1998). [31] | Nepal. National, disaggregated to Mountains region cluster | Mobile camps in permanent contraceptive services (sterilisation) for women, based on Family Health Survey of 1996. | Quality equivalent to hospitals. Camp serves more remote clients however overall there was higher use in the Terai than Mountains and Hills (p<0.05). Regret by 12% in hospital and 10% at camps. | Cross-sectional. Secondary analysis. 817 ever-married women sterilised in camp or hospital | Analysed for confounders and statistical significance. | Moderate |
| **Maternity birthing homes** | Kwast, BE. (1995). [32] | Indonesia. Tanjungsari, West Java | Birthing homes (*polindes*) staffed with a midwife and birthing equipment with radios links to health centres established in a cluster of villages. Also provided health information for women, husbands, leaders and TBAs. | TBA referrals to midwives rose for ANC from 19 to 62% (p<0.001), for delivery from 17 to 36% (p<0.05). Utilisation of SBAs higher in intervention than control; 15 vs 4%. Two of ten *polindes* closed after 2 years due to low retention of midwives. | Cross-sectional. Intervention/ control. Area population of 90,000 | Data collection method not detailed. Limited statistical analysis | Weak |
| **Telemedicine** | Thapa, D. et al. 2010. [33] | Nepal. Myagdi district | Nepal Wireless Networking Project; computers and wireless internet connected villages (approximately 2,000 people) with social, education, economic, healthcare opportunities. | Increased access to and utilisation of specialist doctors through the telemedicine centre. Higher patient satisfaction with better quality care through direct prescribing by, and supervision from, urban doctors to remote health post. | Case study. Qualitative. 2000 population in 2 villages. 40 interviews, FGDs, observations. | Selection bias; snowball method. Wide cross section of respondents and methods. | 8/10 |
| Improving facility and provider performance for quality and satisfaction | | | | | | | |
| **Upgrading** **facilities** | Duke, T. (2000). [34] | Papua New Guinea. Goroka, Eastern Highlands | Upgrade of hospital neonatal care unit - refurbished incubators and warmers, guidelines, in-service training and clinical supervision of nursing staff. | Higher utilisation and quality of care: small increase in admissions in the second year, 44% lower risk of neonatal death (RR 0.56, 95%CI 0.45-0.69) in two-years, largely reduced pneumonia deaths. | Cross-sectional. Before/after.  2414 newborn admissions. | Risk diagnostic inconsistency - changes in staff and definitions. | Moderate |
| **Integrating services at facilities** | Liambila, W et al. (2009). [35] | Kenya. Rural Nyeri and Thika districts | Integration of HIV care with family planning services using an algorithm in counselling sessions for provider initiated HIV test. Compared to standard care of referral for HIV testing. Residential training of 5 days for referral, 9 days for integrated care. | Clients requesting an HIV test increased from 1 to 26%, one third of these had never been tested previously. Integrated care required additional 2-7 minutes per consultation, depending on HIV testing. Integrated care led to more HIV testing; 35% vs 20% (p<0.001) | Cross-sectional. Before/after. Random selection for observations and interviews. 538 pre, 520 post. | No follow-up of HIV test uptake among referred controls. No analysis of site differences | Moderate |
| **Coaching providers for effective communication, responsive and sensitive care** | de Haan, O. (2010a). [36] / de Haan, O. et al (2010b). [37] | Tajikistan, Shahrinav.  Kyrgyzstan, Issyk Kul. | Training health workers with ‘Professional Attitude & Effective Communication’ in parallel to a network of “Parents Schools” to educate clients of reproductive health and services. | FBD users had higher satisfaction and partners attendance increased from 10 to 70%. Quality of care increased through job satisfaction and motivation, reduced harmful practices by 90%, for 30% fewer delivery and caesarean complications. | Cross-sectional. Before/after. Survey of 180 pregnant women, 51 men, 105 providers | Methodology not detailed | Weak |
|  | Kwast, BE. (1995). [32] | Guatemala. Quetzaltenango | Fostering good relationships between formal and informal providers through training TBAs for referrals and facility staff for kindness and respect. Posters at facilities with respectful and inclusive messages. | Perinatal deaths among referrals reduced from 22 to 12% (p<0.05). Early referrals by TBAs for complicated births rose from 26 to 36%, for non-complicated births from 5 to 21%. Referrals by untrained TBAs also rose after training of health staff. | Cross-sectional. Before/after.  761 referrals. | Methodology not detailed. | Weak |
| **Training and supervision** | Brambila, C. et al. (2005). [38] | Guatemala.  Quiché, Quetzaltenango | A distance-learning model for training remote health workers. 40 in-class hours at their peripheral work site over four months through job-aids and peer lessons. | Mmore clients given complete counselling. Women offered family planning up from 7% to 19% (p<0.001) and acceptance rose by 12% (p<0.004) after four months. No significant changes in control. | Controlled trial, Intervention/ control. Before/ after. 2311 women of reproductive age. | Random selection of sites. | Strong |
|  | Leon, FR. et al. (2005). [39] | Guatemala. Quetzaltenango  highlands | The ‘balanced counselling strategy’ - 22 step algorithm and card aides to simplify the client’s task of choosing a contraceptive method and essential information for effective method use. | Uptake by providers was high at 72%. Quality of care improved - receipt of complete services rose from 17 to 42% (p<0.05) (no change in controls). Uptake rose from 31 to 34 clients per day. | Quasi- experimental trial. Intervention/ control. Before/ after. | Unmatched. | Moderate |
|  | Vernon, R. et al. (1994). [40] | Guatemala. Areas of  Baja Verapaz, El Progreso, Jutiapa, San Marcos, Quiché | Traditional staff supervision of 2 annual supervisor visits tested against ‘indirect method’ of supervisor field visit plus 1-day district-level meeting, and ‘self-assessment’ of supervisor field visit plus 2-day self-assessment. | CPR increased by 9.7% with indirect supervision, 6.5% in self-assessment but reduced by 22% in control. Staff prefer indirect supervision; 96% participation vs 77% for self-assessment. Both new methods more cost-effective than traditional. | Controlled trial  Survey of 71 providers, 327 women who use contraception. | Unmatched. | Strong |
| Financial initiatives to ease the burden of service fees and costs of care-seeking | | | | | | | |
| **Free maternity care services** | Witter, S. et al. (2011). [41] | Nepal. 6 districts including Jumla. | Free maternity care services through National policy, reimbursing facilities with Government funds per facility-based birth. | FBD for normal births rose by 19%, for complications by 15%, for caesarean by 18%. Perceived reduced delays in receiving care. Patients still charged fees at some public facilities due to loss of revenue. | Cross-sectional.  Before/after.  Records of 22 facilities, 50 health manager interviews | No statistical analysis. | Weak |
| **Financial stimulants** | Powell-Jackson. et al. (2008). [42] / Powell-Jackson. (2009). [43] | Nepal. 10 districts including Jumla and Sankhuwasabha | Three-fold financing initiative of Nepal’s Safe Delivery Incentive Programme; conditional cash transfers for demand generation, cash incentives for providers and compensation to facilities, and free FBD for women. | FBD 24% more likely if exposed to the program. Promotion difficult in sparsely populated mountains. Incentive met only 30-50% of travel costs in the Mountains. Guidelines poorly understood or obeyed. Funds shortages and delays cause distrust. | Cross-sectional.  Before/after.  50 facilities, 40 health managers, 8 FGDs, survey of 5,513 women | Two-stage sampling, PSU and random selection. Wide cross section of data sources. | 10/10 |
| Community input for demand motivation and culturally-appropriate health services | | | | | | | |
| **Community planning** | Kwast, BE (1995). [32] | Bolivia. Rural Inquisivi | Structured women's group problem solving. Training women and their spouses for safe home birth, family planning and linking to hospitals. See also O’Rourke. | CPR rose by 27%, early breastfeeding from 25 to 57%, prenatal check-up increased from 45 to 77%. 27% of homebirths used the CDKs which were new to the area. | Cross-sectional  Before/after.  Intervention/control. Total 761 births. | Very varied sample sizes. Confounders not assessed. | Weak |
|  | O'Rourke, K. et al. (1998). [45] | Bolivia. Rural Inquisivi | Warmi Project; structured women’s groups; 1) problem definition, 2) planning, 3) implementation, and 4) participatory evaluation. Groups promoted birth safety, family planning to women and men, with NGO developed women's health card, booklets and radio programs. | Perinatal mortality decreased from 117 to 43 deaths per 1000 live births (p<0.001). Increases in participation in organisations from 7% to 54% (p<0.001), ANC from 49% to 64% (p=0.009). | Case-control study for mortality.  Before/after. 409 mothers | Matched, controls to cases 2:1.  Strong effect | Moderate |
|  | Rios R, et al. (2007). [46] | Bolivia. Rural departments of Chuquisaca, La Paz, Potosi, Tarija. | Local women trained as health promoters host three hour workshops with community members discussing reproductive and maternal health issues, cultural beliefs and local responses. | 73% reported improved attitude and an intention to use family planning. 9.7% increase in contraceptive use compared with previous year. | Cross-sectional  Before/after.  Sample size not specified. | Methodology not detailed. Very limited data and analysis. | Weak |
|  | Ashwell, HE (2009). [47] | Papua New Guinea. Nipa –Kutubu, Kikori, Rai Coast, Mul-Baiyer, Sumkar, Huon, Markham, Nawae, Ijivitari, Kompiam. | Community Action and Participation Program to create ‘wellbeing villages’ and increase health service use. Promotion of leadership, visual tools for community education, promotion of positive social behaviour, linked village health volunteers with health workers. | Increased use of RMNCH services in 53% of communities while 26% were 'moderately successful', and 21% improved very little. Success was associated with a health-motivated person acting as a catalyst for change, and empowered leadership. | Cross-sectional  Before/after  175 interviews with health workers, managers, men, women, 77 FGDs. Health records | Targeted sampling. Weak baseline for comparison  Proportion changes are subjective | 8.5/10 |
|  | Aarons, A (1983). [48] | Papua New Guinea. Southern Highlands | United planning between school teachers, health workers, nutritionist, agricultural workers, and community leaders for community-wide action for child health beginning with a three-day workshop. | Increased child health education in school curriculum, kitchen gardens created, increase sanitation & hygiene through hand-washing and construction of pit latrines. Service use increase but not quantified. | Case studies. Qualitative. Before/after | Methodology not detailed | 4/10 |
| **Engaging traditional healers** | Hoff, W. (1992). [49] / Oswald, I (1983). [50] | Nepal, Taplejung | Linking traditional healers with formal health workers and defining their roles through training in health education and promotion, in Nepal specifically targeting tuberculosis, leprosy, childhood diarrhoea and malnutrition. Revisits every 2 months. | Increase use of rural clinics after training traditional healers, up to double in some sites. Faith-healers are culturally-appropriate sources of family planning messages. Improved attitudes, knowledge, and behaviour of both traditional and formal providers. Defined roles were essential. | Controlled trial. Intervention/ control. Before/ after. 4 health posts, referral records, 22 traditional healers | Very small sample. Risk of selection bias –most popular and prominent traditional healers. | Moderate |
| Knowledge and awareness-raising to motivate care-seeking | | | | | | | |
| **Awareness-raising** | Erulkar, A. et al. (2009). [51] | Ethopia. Rural Amhara Province | A program of three components: dialogue including men and women of all ages for problem solving strategies, mentor groups for female youth, literacy and livelihood skills training for young women. | After two years, school enrolment for girls, 10-14 years, rose from 71 to 96% (p<0.05), contraceptive use among sexually active girls rose from 43% to 74% (p>0.001). | Quasi-experimental Intervention/ control. Before/ after. 1386 households. | Controlled for confounders  Strong effect | Strong |
|  | Natoli, LA. et al. (2008). [52] | Ethiopia. Amhara region | Teaching of men, then counselled couples together to reduce harmful traditional practices such as female genital mutilation (FGM), traditional birth rituals, early marriage, high fertility. Harmful practices disciplined by the local Council. | Attitude shift to disapproval of FGM, increased contraceptive use, reduced total fertility rate. A traditional stretcher is now ready-made in advance for referral transport. Male involvement as community health messengers was key for change. | Cross-sectional.  96 FGD participants, 8 interviews; TBAs, women, men, students, health workers, leaders. | Wide cross section of respondents | 10/10 |
|  | Shankar, A. et al. (2009). [53] | Indonesia Lombok | Community facilitators to liaise between pregnant women and health workers proving information to promote iron-folic acid / multiple micronutrient supplementation and SBA utilisation. | High compliance with supplementation at 85%, SBA rose from 35 to 53% (p<0.001). Community facilitators with higher knowledge, concern and thoroughness linked with 33% reduction in early infant mortality (RR 0.67, 0.49-0.92, p=0.011) | Double-blind RCT.  Intervention/ control. Before/ after. 31,290 pregnant women | Large sample, strong design | Strong |
| **Information tools** | Black, TR. et al (1976). [54] | Kenya. Rural Meru and Kirinyaga districts. | Social marketing for condom use. Baseline investigation informed product appearance and price. Messages of health and economic benefits of birth-spacing through leaflets, radio Q&A and announcements, village cinema shows, shops displays. Mobile education unit with free samples. | FP use increased from 21 to 35% (p = 0.01) - almost entirely condom use. FP awareness increased from 64 to 81% (p=0.01). No change in control. Stocked at 56% of the district's stores after 12 months. Engaging retailers of high importance as they are key sources of consumer information. | Controlled trial. Intervention/ control. Before/ after. Interviews; 60 retailers, 1005 adults in Meru, 700 in Kirinyaga, | Strong design, random sampling | Moderate |
|  | Kwast, BE (1995). [32] | Indonesia. Highlands of East Java | Women volunteers trained to use colour coded score card to grade each pregnant woman’s risk based on clinical history and symptoms (no physical examination). Women at high or very high risk strongly advised to have a FBD. | Home visits were repeated by PKKs for almost all pregnancies. Poor compliance with advice; only 10% of high risk and 27% of very high risk women had FBD. No verification of the quality of PKKs’ work or accuracy of their risk classifications. | Controlled trial.  Intervention/ control  Before/after. 8375 births | No statistical analysis shown | Moderate |

**Abbreviations**: ANC – antenatal checkup, CBRHW – community-based reproductive health worker, CEOC – comprehensive emergency obstetric care, CHW – community health worker, CPR – contraceptive prevalence rate, FBD – facility-based delivery, FCHV – female community health volunteer, FGD – focus group discussion, FP – family planning, HEW – health extension workers, IUD - intrauterine contraceptive device, LHW – lady health worker, MMR – maternal mortality ratio, NPC – non-physician clinicians, OR – odds ratio, PNC – post-partum check-up, RMNCH – reproductive, maternal, newborn and child health, RR – relative risk, SBA – skilled birth attendant, TBA – traditional birth attendant.
